# Tryptophan, a non-canonical melanin precursor: New L-tryptophan based melanin production by *Rubrivivax benzoatilyticus* JA2

**DOI:** 10.1038/s41598-020-65803-6

**Published:** 2020-06-02

**Authors:** Shabbir Ahmad, Mujahid Mohammed, Lakshmi Prasuna Mekala, Sasikala Chintalapati, Venkata Ramana Chintalapati

**Affiliations:** 1 Department of Plant Sciences, School of Life Sciences, University of Hyderabad, Hyderabad, 500046 India; 2 Centre for Environment, IST, JNT University, Hyderabad, 500 085 India; 3 Present Address: Department of Botany, Bharathidasan Government College for Women, Puducherry, U.T. – 605003 India; 4 Present Address: Department of Plant Sciences, Avvaiyar Government College for Women, Karaikal Puducherry, U.T 609602 India

**Keywords:** Microbiology, Physiology

## Abstract

Melanins are chemically diverse ubiquitous pigments found across the life forms synthesized *via* different biochemical pathways mainly from L-tyrosine or acetyl CoA. Though few reports suggest the possibility of tryptophan-based melanin synthesis, however, such tryptophan-based melanin and its biosynthesis remained a biochemical riddle. Here we report tryptophan-based melanin production by bacterium, *Rubrivivax benzoatilyticus* JA2. Aerobic cultures of strain JA2 produced brown pigment when grown on L-tryptophan-containing media. Purified pigment showed typical physico-chemical properties of melanin. Further, extensive spectroscopic studies revealed that pigment is an amorphous, indole-type polymer with stable free radical centers. Further, hydrolysis of the brown pigment revealed the presence of indole moiety, confirming the indolic nature of the pigment. Demonstration of *in vitro* and *in vivo* pigment synthesis directly from L-tryptophan or hydroxytryptophan confirms tryptophan-based melanin synthesis in strain JA2. Interestingly, canonical melanin biosynthetic inhibitors did not affect the pigment synthesis indicating possible non-canonical tryptophan-based melanin biosynthesis in strain JA2. Further, the exometabolite profiling and precursor feeding studies suggests that L-tryptophan converted to hydroxytryptophan/hydroxyindoles and their subsequent polymerization lead to the formation of melanin. The current study sheds light on biosynthetic diversity of melanins and L-tryptophan can be a potential precursor for melanin synthesis in life forms.

## Introduction

Microorganisms are prolific producers of a diverse array of pigments and these pigments are produced under different physiological conditions. The pigments are structurally diverse ranging from simple low molecular organic compound to complex macromolecules such as melanins^1^. Melanins are structurally complex, ubiquitous heterogenous polymeric pigments found in all biological systems^2–5^. Melanins are enigmatic biopolymers formed by oxidative polymerization of phenolic and hydroxyindole derivatives^3,6^. Melanins display different colors such as black, brown to yellow and are highly hydrophobic amorphous polymers^2,3,6^. Melanins are negatively charged^3^ and contain stable free radicals, a characteristic feature of melanins and are highly resistant to degradation^4,5,7^.

Melanins display remarkable chemical diversity and are classified as eumelanin, pheomelanin, pyomelanin, allomelanin, neuromelanin and DHN melanin based on the color and chemical composition of the melanin^3,8,9^. Eumelanin, pheomelanin, pyomelanin, and neuromelanin are synthesized from L-tyrosine^3,8^ whereas, DHN melanins are synthesized from acetyl CoA or malonyl CoA as a precursor^7^. Eumelanins contain nitrogen, pheomelanin contains both nitrogen and sulfur while allomelanin or DHN melanin contains neither of them^3,8^. Diverse life forms synthesize different melanins, for example, several groups of fungi produce mainly nitrogen-free DHN melanin as well as nitrogenous DOPA melanin^6,7,10^. Eumelanins are mainly found in all animals imparting different colors and few microorganisms also produce eumelanin^2,3,8^. Except for the DHN melanin, all other types of melanins are synthesized from, tyrosine/phenylalanine. On the other hand, DHN melanins are synthesized mainly from acetyl CoA/malonyl CoA using the polyketide synthase enzyme system^7,8,10^. Though melanins are chemically diverse their biosynthetic origin is fairly simple starts from tyrosine or acetyl CoA as a precursor^8,11^. However, a recent study revealed a non-canonical Asp-melanin biosynthesis in *Aspergillus terreus* indicating melanin biosynthetic diversity in life forms^12^. Similarly, few *in vivo* studies in animal models^13,14^ and *in vitro* oxidation studies suggested tryptophan can be a potential precursor for melanin synthesis^14,15^. However, tryptophan-based (named as Trp-melanin) melanins are neither characterized nor their biosynthetic pathway is identified and more so there are no reports of Trp-melanins in microorganisms.

Anoxygenic photosynthetic bacteria are metabolically versatile yet less explored group of bacteria capable of producing different biomolecules such as carotenoid pigments^16,17^ and melanin using aromatic amino acids as precursors^18^. *Rubrivivax benzoatilyticus* JA2 is one such a photosynthetic bacterium with remarkable aromatic compound biotransformation abililty^19–22^. Studies on aromatic compound metabolism by strain JA2 revealed the production of several value-added compounds^22,23^ and multiple catabolic pathways^22,24^. Recently we reported pyomelanin production by aerobic cultures of strain JA2 and genomic and metabolic insights revealed pyomelanin biosynthetic pathway in strain JA2^18^ Employing the newly developed metabolite-centric approach we identified anthocyanin-like pigment production in phenylalanine-amended aerobic cultures of strain JA2^19^. Our recent studies on aerobic aromatic metabolism of strain JA2 revealed new biomolecules and metabolic pathways^18,19,24^. Similarly, while working on aerobic L-tryptophan metabolism in strain JA2 surprisingly we found melanin-like pigment synthesis in aerobic tryptophan amended cultures. In the present study, we report a tryptophan-based melanin production for the first time in a microorganism and characterized the novel melanin produced by strain JA2. The study also suggests a possible non-canonical route of tryptophan-based melanin (Trp-melanin) synthesis.

## Results

### Growth, L-Tryptophan utilization, and pigment production

**Figure 1:**
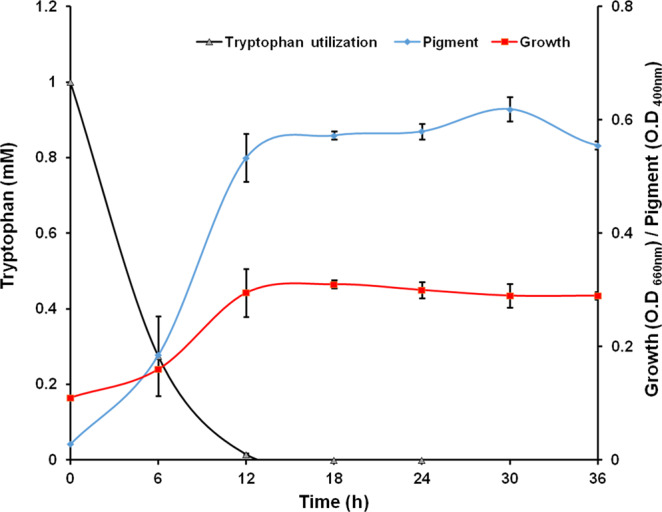
Growth, tryptophan utilization and brown pigment production by *Rubrivivax benzoatilyticus* JA2 under aerobic conditions. Values are the mean ± standard deviation of two biological replicates.

**Figure 2:**
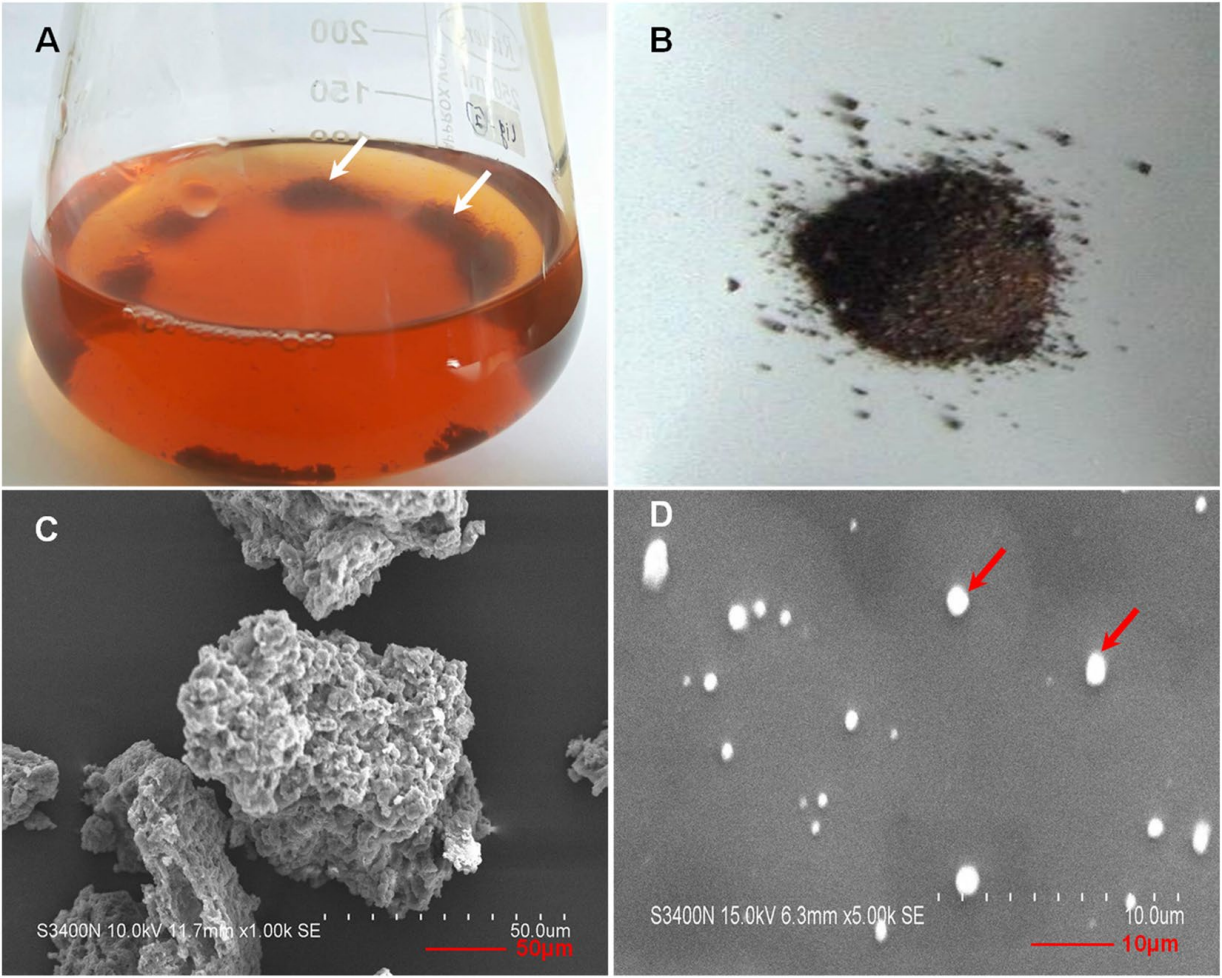
Image showing the brown pigment in acidified culture supernatant obtained from tryptophan-amended aerobic culture of strain JA2 (**A**), Dry purified pigment (**B**), SEM micrograph showing aggregated granules of pigments (**C**) and dispersed individual spherical granules of pigment (**D**).

Strain JA2 could grow on L-tryptophan as a nitrogen source under aerobic conditions and utilized 90% of L-tryptophan within 12 h of incubation (Fig. 1). Strain JA2 produced brown pigment with concomitant utilization of L-tryptophan and pigment production was higher at 12 h wherein the maximum amount of tryptophan was utilized (Fig. 1). The pigment was produced only in tryptophan-containing aerobic cultures while no pigment was observed in L-tryptophan-amended anaerobic as well as control (without tryptophan) aerobic cultures (Fig. S1). The pigment produced only in L-tryptophan-containing media inoculated with strain JA2 and pigment was not formed in un-inoculated tryptophan-containing media. The pigment produced by strain JA2 was purified from acidified culture supernatants of L-tryptophan-amended aerobic cultures and upon acidification, the pigment settled as a brown precipitate (Fig. 2A). The dried pure pigment appeared as dark brown (Fig. 2B) and the yield of the pigment was 33 ± 3 mg dry weight per 0.5 liters.

### Physicochemical properties of purified brown pigment

The purified pigment was soluble only in alkaline solution (1 M NaOH) and insoluble in organic solvents (hexane, chloroform, acetone, ethyl acetate, ethanol, benzene) as well as water. Pigment is insoluble in neutral buffers and sparingly soluble in alkaline buffers (pH:10–12) (Table S1). The pigment readily precipitated in acidic conditions (5 M HCl), bleached when treated with oxidizing agents like H_2_O_2_ and NaOCl (Table S1). The pigment gave a positive reaction to the polyphenol test by forming flocculent brown precipitate reacting with FeCl_3_ (Table S1). Pigment gave positive to ammonical silver nitrate test and Na_2_S_2_O_4_ addition decolorized the pigment, upon adding potassium ferricyanide colorless pigment turned brown. The purified brown pigment was positive to all the characteristic chemical tests used to identify melanins^25^ (Table S1).

### Characterization of brown pigment revealed that the pigment is melanin

**Figure 3:**
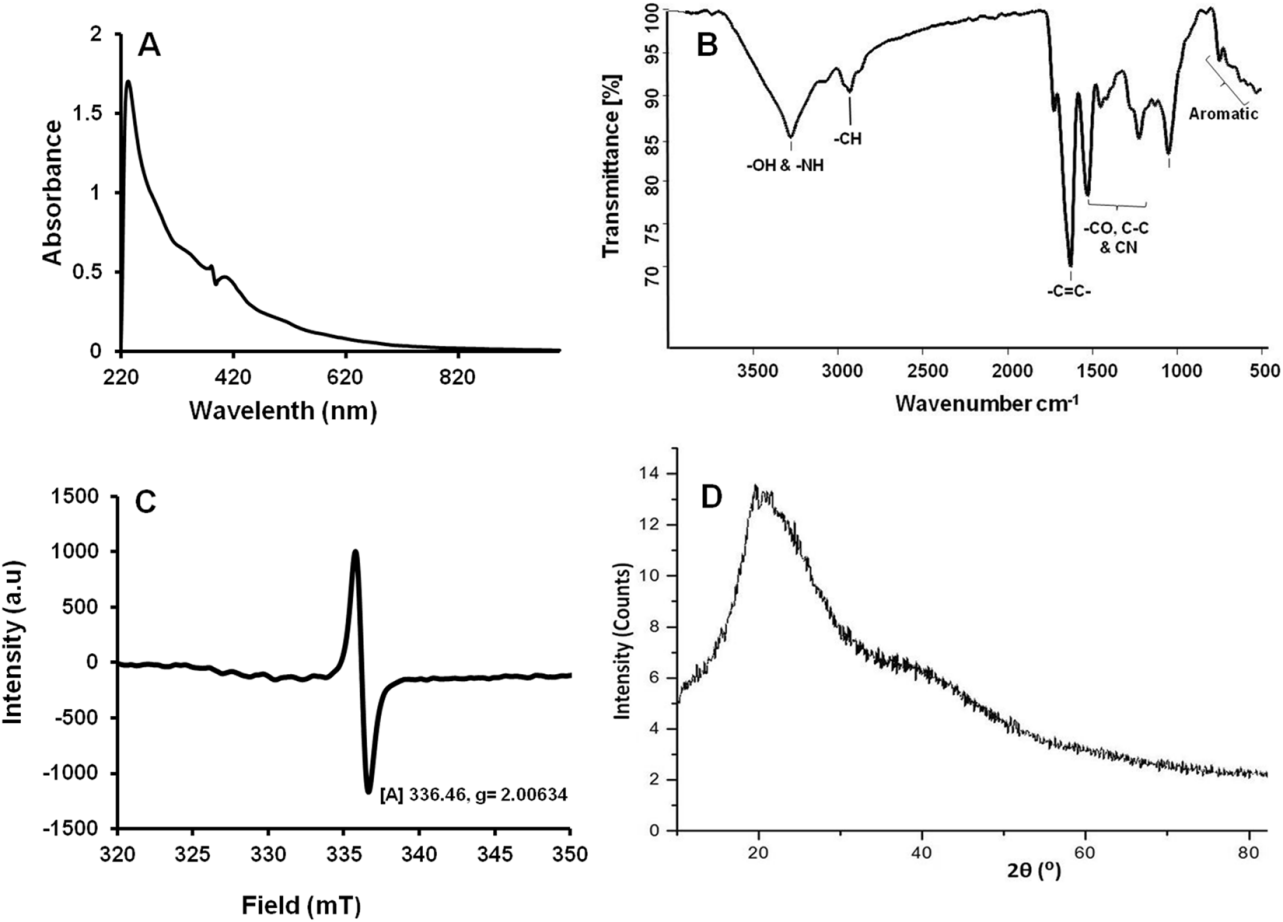
Spectroscopic characterization of purified brown pigment. (**A**) UV-Visible spectrum; (**B**) FTIR spectrum; (**C**) ESR spectrum; (**D**) X-Ray Diffraction spectrum.

Scanning electron micrographs of the purified brown pigment revealed unorganized aggregated spherical granules of pigment (Fig. 2C) while the SEM micrograph of the sonicated sample showed dispersed typical sphere-like structures with the homogenous smooth surface characteristic of typical melanin^26^ (Fig. 2D). The UV-Visible spectrum of purified pigment showed a broad absorption spectrum from UV to the visible region and the absorption was higher in the UV compared to visible region (Fig. 3A). Absorption of brown pigment decreased progressively with increasing wavelength showing a linear correlation between absorption and wavelength (Fig. S2), a characteristic feature of melanins^26,27^. Elemental analysis of purified brown pigment revealed the presence of C, H, N, O, and no sulfur was detected. Pigment consisted of carbon-46.73%-, hydrogen-5.26%, nitrogen-9.16% and oxygen-38.38% and high C/H ratio (8.9%) of brown pigment indicates the presence of many fused aromatic structures in the pigment^26,28^. Presence of high nitrogen content (9%) and absence of sulfur moiety indicates that brown pigment is a nitrogen-containing pigment.

FTIR spectrum of the purified pigment exhibited the absorption bands typical to a functional moiety of melanin. The broad band at 3276 cm^−1^ can be assigned to the –OH and –NH stretching vibrations and the bands at 2928–2802 cm^−1^ ascribed to CH_3_, -CH_2_ stretching (Fig. 3B). The band at 1723 cm^−1^ assigned to C = O stretching vibrations from carbonyl, carboxyl, ketone or quinone groups present in the pigment^29^. Strong absorption at 1626 cm^−1^ can be assigned to C = C stretching vibrations of aromatic/pyrrole moiety^29^ (Fig. 3B). The band at 1526 cm^−1^ can be designated to the N-H bending along with the band at 1414 cm^−1^ (C-N stretching) suggests the presence of indole/pyrrolic functional groups in the structure of the pigment^27,29^. Absorption at 1220 cm^−1^, indicative of –C-OH group of a phenolic or indolic moiety of pigment^30^ (Fig. 3B). The band at 1048 cm^−1^ can be ascribed to the CH- in-plane or CH- out plane deformations and abruption below 700 cm^−1^ corresponds to out of plane carbon-hydrogen bending of the aromatic moiety^30^. The IR spectroscopic properties of the pigment of strain JA2 closely correlated with eumelanin^2,5,30^.

Electron Spin Resonance (ESR) analysis of purified brown pigment revealed the singlet ESR signal at 336.46 mT corresponding to a g-value of 2.00634 (Fig. 3C). Singlet ESR signal observed in the ESR spectrum of brown pigment indicates the presence of stable free-radicals (paramagnetic) a characteristic feature used to identify melanins^7,27^. X-ray diffraction analysis of the purified pigment showed a broad diffraction peak around 2θ = 10–50° suggestive of the amorphous nature of the pigment (Fig. 3D) and this in agreement with previous reports on melanins^18^. Amorphous compounds like melanins which do not have any regular repeating units display broad diffraction features^18^.

### ^13^C and ^15^N solid-state NMR studies

**Figure 4:**
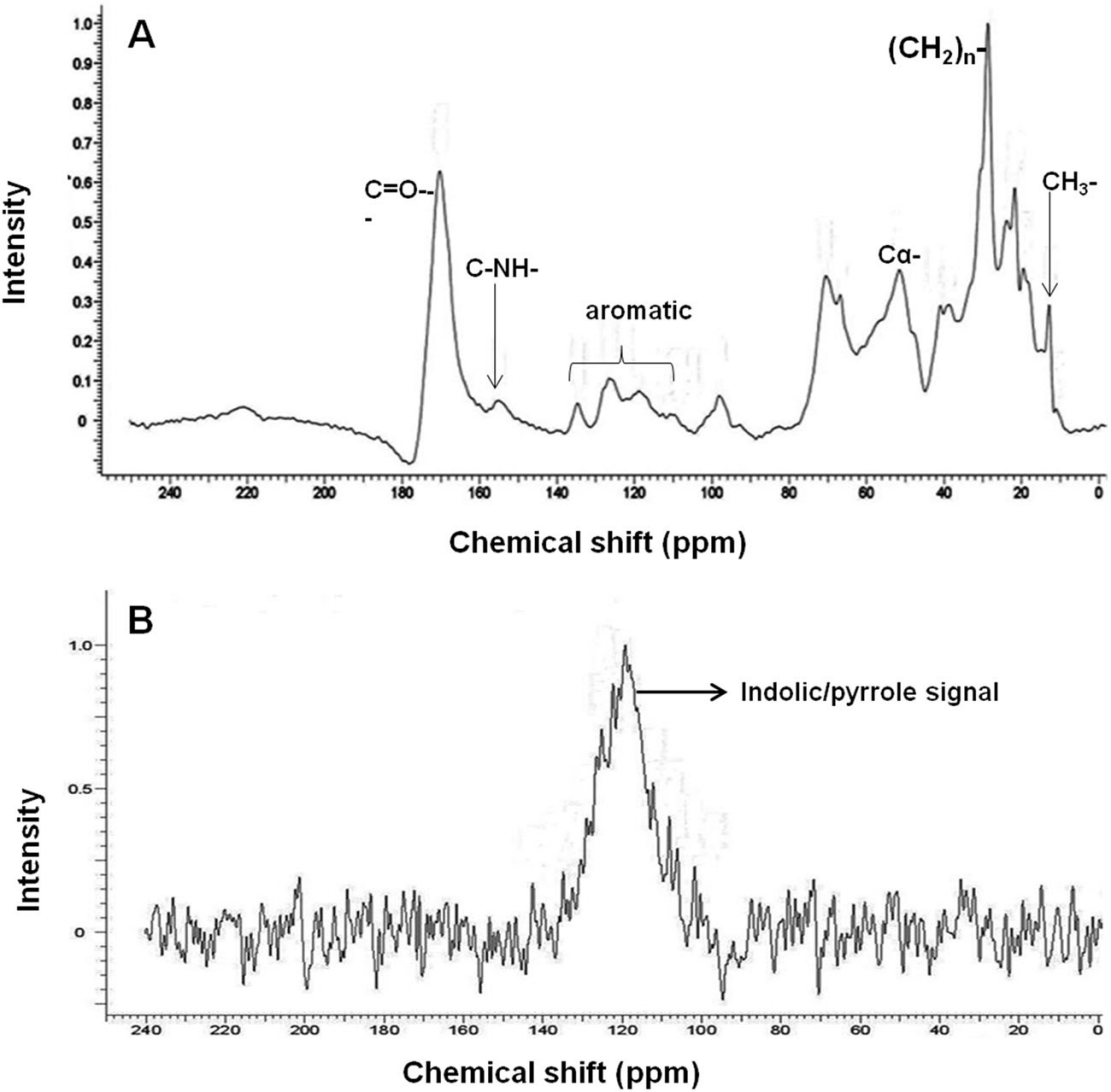
Nuclear Magnetic Resonance spectra of purified brown pigment. (**A**) Solid-state CPMAS ^13^C and (**B**) ^15^N NMR spectra.

The ^13^C solid-state NMR of purified brown pigment showed the intense signal at 160–180 ppm in carboxyl region is usually designated as carbonyl carbon of carboxylates, and quinones possibly associated with a pigment (Fig. 4A)^2,31,32^. Chemical shifts from 110–150 ppm correspond to aromatic carbons and 100–134 ppm with a broad and weak signal at 126, 110 ppm designated to aromatic carbons of indole/pyrrole moiety^2,31,32^. Peak around 156 ppm indicates the presence of C-NH- group, possibly of pyrrole/indole moiety^2^. The intense signal from 10–45 ppm is due to side-chain aliphatic carbons correspond to methyl, methylene and methine groups (Fig. 4A)^2,31,32^. Chemical shifts range from 45–60 ppm can be assigned to backbone carbon of α-C, and β-carbons. The ^13^C solid NMR spectrum of the brown pigment is similar to that of eumelanin spectrum^2,31,32^.

The solid-state ^15^N NMR CP/MAS spectral studies were carried out to further confirm the presence of the nitrogen in the brown pigment. The ^15^N NMR spectrum showed an intense broad chemical shift ranging from 110–140 ppm which is consistent with a chemical shift of the nitrogen atom of indole/pyrrole moiety^31,33^ (Fig. 4B). The ^15^N spectrum of brown pigment is similar to that of the ^15^N spectrum of indole-type Sepia and human hair melanin^31^.

### Effect of canonical melanin inhibitors on brown pigment formation

**Figure 5:**
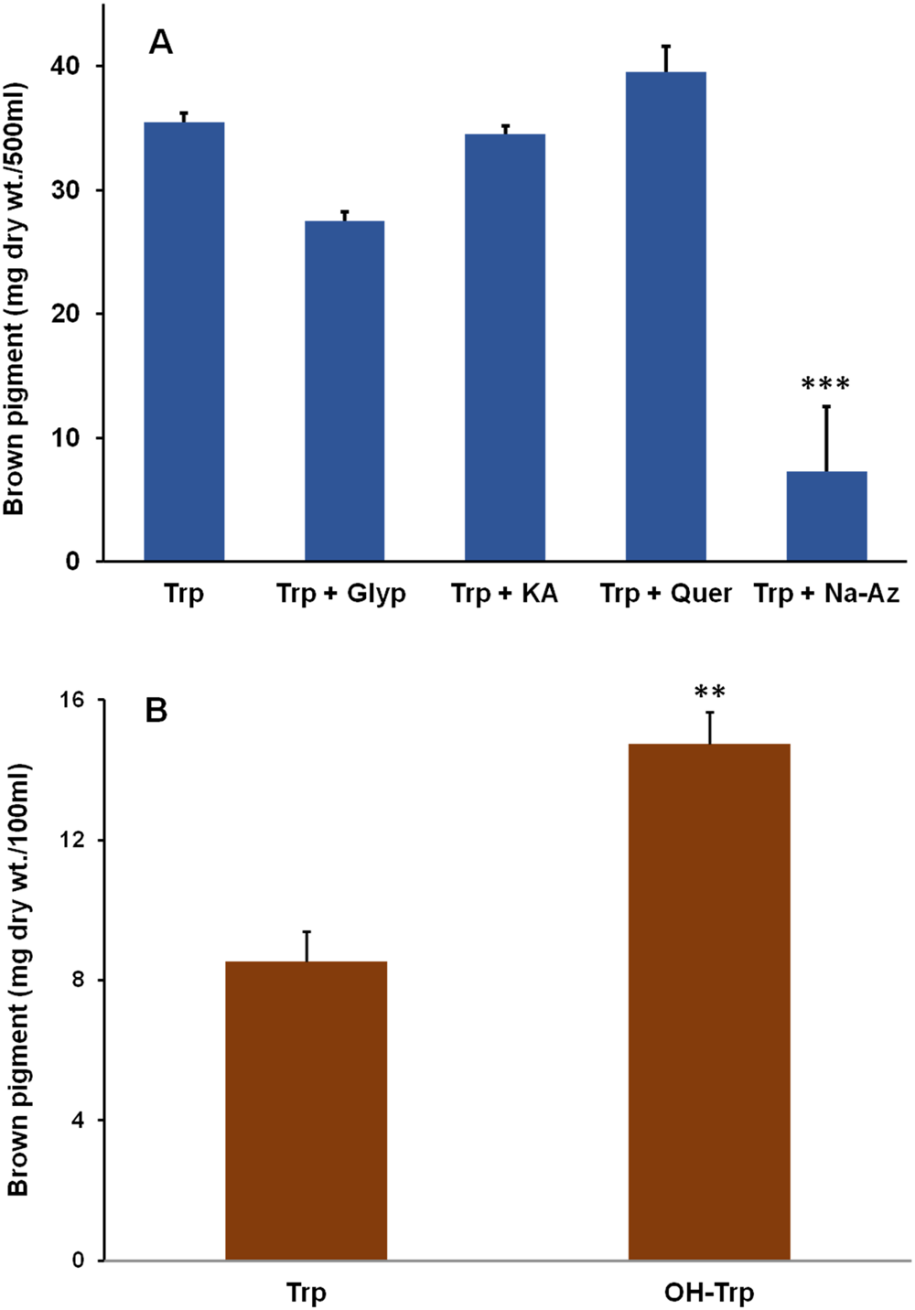
Brown pigment production by strain JA2 in the presence of various inhibitors and substrates. (**A**) Effect of canonical melanin specific biosynthetic pathway inhibitors on brown pigment production under tryptophan-amended aerobic conditions. (**B**) Brown pigment production by strain JA2 grown in the presence of tryptophan and 5-hydroxytryptophan. Values are the mean ± standard deviation of three biological replicates. **P- value <0.005; ***P-val <0.0005 compared to Trp supplemented cultures, calculated using unpaired t-test. Trp, tryptophan; OH-trp, hydroxytryptophan; Glyph, glyphosate: KA, kojic acid; Quer, quercetin; Na-Az, sodium azide.

Pigment production by strain JA2 was monitored in the presence of different inhibitors of known melanin biosynthetic pathways. Glyphosate, an inhibitor of tyrosine dependent melanin synthesis^11^, as well as quercetin, kojic acid, a eumelanin inhibitor^2^ has no effect on pigment production (Fig. 5A). Similarly, pigment production was unaffected in the presence of sulcotrione (visual observation), a pyomelanin inhibitor^11,18^. Interestingly sodium-azide, a laccase specific inhibitor significantly inhibited the pigment production (Fig. 5A).

### HRMS based exometabolite profiling of Trp-fed aerobic cultures

LCMS based exometabolite profiling was carried out to identify the indole derivatives of aerobic tryptophan metabolism and potential immediate precursor/intermediates of pigment synthesis. Typical total ion chromatogram of methanolic extracts of aerobic Trp-amended culture supernatants represented here showing the elution profile of the tryptophan-derived metabolites (Fig. S3). LCMS analysis of a methanolic extract of aerobic Trp culture supernatants revealed metabolite showing identical UV-Visible profile, Rt 7.9 mins having molecular ion mass of 219.0778 [M^-^] corresponding to 5-hydroxytryptophan (Table 1). Metabolite with Rt 14.3 mins also showed identical UV-visible spectrum and molecular ion mass of 190.0509 [M^-^] correspond to 5-hydroxyindole-3-acetic acid (Table 1). Subsequently, metabolites with Rt 7.9 and 14.3 mins were confirmed as 5-hydroxytryptophan and 5-hydroxyindole-3-acetic acid by co-eluting with authentic standards. Metabolites with Rt 10.9, 15.5 and 16.3 mins having molecular ion masses of 206.04, 218.04, and 147.06[M^-^], were tentatively identified as 5,6-dihydroxyindole-3-acetic acid (DHIAA), 5-hydroxyindole-3-pyruvate (HIPy), and 5-hydroxy-methyl-indole (HMI) respectively based on mass spectra search against database hit and UV-visible profiles.

**Table 1:** Tryptophan derived metabolites detected in tryptophan-amended aerobic cultures of strain JA2.

S. no.	R_t_ (min)	Generated Mol. formula	Exact Mass	m/z [M^-^]	Mass accuracy (ppm)	UV-visible Absorbance (nm)	Identification (Confirmed/ Tentative)
1	*7.9*	*C*_11_*H*_12_*N*_2_*O*_3_	***220***	*219.0778*	*0.75*	***275***	*5-Hydroxytryptophan*
2	10.9	C_11_H_9_NO_4_	207	206.0458	0.86	270, 280, 288	5,6-dihydroxyindole-3-acetic acid
3	*14.3*	*C*_10_*H*_9_*NO*_3_	***191***	*190.0509*	*0.34*	*275, 300*	*5-hydroxyindole-3-acetic acid*
4	15.5	C_11_H_9_NO_4_	219	218.0456	1.42	260, 292	5-Hydroxyindole-3- pyruvate
5	16.3	C_9_H_9_NO	147	146.0609	1.36	290	5-hydroxy-methyl-indole

Metabolites in italics are confirmed by authentic standards.

### Brown pigment formation from L-tryptophan and 5-hydroxytryptophan

As we found 5-hydroxytryptophan (OH-Trp) in culture supernatants of Trp-amended cultures, we checked the pigment production in the presence of Trp and OH-Trp in strain JA2. Interestingly, strain JA2 produced intense brown pigment in OH-Trp containing media (Fig. 5B) while no pigment was formed in uninoculated OH-Trp containing media. The pigment production was significantly higher in the presence of OH-Trp compared to tryptophan (Fig. 5B). Pigment purified from OH-Trp-containing cultures showed similar physicochemical properties and UV-Visible profile with that of pigment obtained from Trp cultures (data not shown).

### *In vitro* pigment formation by strain JA2

**Figure 6:**
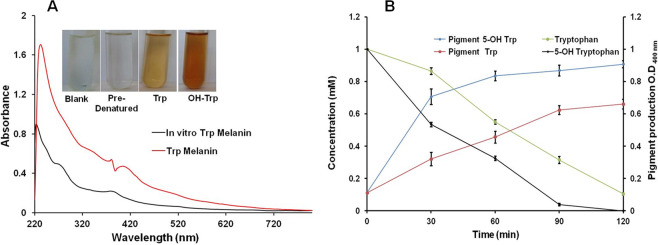
*In vitro* formation of brown pigment in the presence of tryptophan and 5-hydroxytryptophan. (**A**) *In vitro* pigment formation. (**B**) Time-dependent *in vitro* pigment formation. Figure shows UV spectra of pigment obtained from *in vivo* and *in vitro* assays from Trp, Inserts shows *in vitro* pigment formation.

Strain JA2 showed *in vivo* pigment formation from Trp as well as OH-Trp containing media under aerobic conditions. Here we tried to replicate the pigment formation *in vitro* by exogenously supplying L-Trp and OH-Trp to cell-free extracts obtained from tryptophan supplied aerobic cultures. *In vitro* study revealed the pigment formation in L-Trp and OH-Trp supplied cell-free reactions while pigment formation was not observed in pre-denatured cell-free extracts or reaction mixture without Trp/OH-Trp (Fig. 6A). Pigment intensity was high in OH-Trp compared to L-Trp supplemented *in vitro* assays and purified *in vitro* pigment displayed similar UV-Visible profile (Fig. 6A). *In vitro* studies on pigment formation revealed simultaneous consumption of L-Trp or OH-Trp and formation of pigment over a period of time. Further, the pigment content was high in case of OH-Trp compared to L-Trp (Fig. 6B).

### Brown pigment hydrolysis and chemical oxidation confirms the indolic nature of the polymer

Chemical hydrolysis of the brown pigment was performed to confirm the presence of indole derivatives in brown pigment. TLC analysis and indole specific staining of hydrolyzed products obtained from tryptophan (Trp) and hydroxytryptophan (OH-Trp) brown pigments revealed the identical staining pattern in the form of two purple bands indicative of indole moiety (Fig. S4). While staining was not observed in case of unhydrolyzed fractions (methanolic washout) of Trp and OH-Trp pigments indicating that indoles were indeed derived from hydrolyzed brown pigments (Fig. S4). Further chemical oxidation of brown pigment and subsequent HR-LCMS analysis indicated presence of molecular ion mass of 198.0 [M-H] and 241.8 [M-H] corresponding to the pyrrole-2,3,5-tricarboxylic acid(PTCA) and pyrrole-2,3,4,5-tetracarboxylic acid (PTeCA), respectively (Fig. S5). Further PTCA and PTeCA, were confirmed by mass spectral fragmentation pattern, UV spectra and co-elution with authentic standard (only PTCA). PTCA and PTeCA are oxidative products of 5,6-dihydroxyindole-2-carboxylic acid (DHICA)^5,9,34^. Taken together the indole staining of hydrolysed brown pigment and chemical oxidation confirms that brown pigment is indeed an indolic in nature.

## Discussion

Melanins are chemically diverse pigments mainly synthesized from two precursors acetyl-CoA (DHN melanin) or tyrosine (eumelanin, pheomelanin, allomelanin, pyomelanin) *via* diverse pathways^2,8^. Here we identified tryptophan-based melanin (Trp-melanin) synthesis in a bacterium and characterized the pigment and study suggested possible non-canonical Trp-based melanin synthesis. *Rubrivivax benzoatilyticus* JA2 is a metabolically versatile photosynthetic betaproteobacterium and it has remarkable aromatic metabolism evident from a number of new biomolecules^16,35,36^, catabolic pathways^19,20,22–24,35,37^, and enzymes reported^24,36^. Recently we designed a new metabolite-centric secondary metabolite mining strategy and employing this tool we successfully demonstrated novel anthocyanin–like pigment production in phenylalanine-amended aerobic cultures of strain JA2^19^. Similarly, while working on aerobic metabolism of L-tryptophan, serendipitously we found brown pigment production in strain JA2. Interestingly, only Trp-amended aerobic cultures of strain JA2 produced brown pigment with concomitant utilization of Trp and this suggests that pigment synthesis is Trp-dependent oxidative metabolic process.

Surprisingly, the purified pigment displayed the characteristic properties of melanin such as alkaline solubility, insoluble in organic solvents, positive to polyphenol test and decolorizing to oxidizing agent^25^ indicating that the brown pigment is melanin (Table S1). Furthermore purified brown pigment showed typical UV-Visible profile of melanin; higher UV absorption and absorbing all the wavelengths in the visible region further confirms that the pigment is melanin (Fig. 3A). Melanins are amorphous polymers with stable free radical centers^7,27^, in agreement with this, broad diffraction peak and singlet ESR signal displayed by the brown pigment (Fig. 3C,D) of strain JA2 strongly suggests it is an amorphous pigment having free radical centers. Physico-chemical and spectroscopic studies unequivocally confirm that the brown pigment is indeed a melanin. Melanins are known to be synthesized during the oxidative metabolism of phenylalanine/tyrosine^8,18,24,33^, in contrast, here we report oxidative metabolism of tryptophan leading to Trp-melanin formation in strain JA2. Further, the high nitrogen content (9%) and C/H ratio (8.9%) (typical of aromatic polymers)^26,28^ revealed by elemental analysis of brown pigment indicate that the pigment is a nitrogen-containing aromatic polymer.

This is further supported by IR, NMR analysis wherein brown pigment showed characteristic signals of hydroxy, indolic/pyrrolic and aliphatic moieties. Moreover, a broad signal in ^15^N NMR of brown pigment confirms the presence of pyrrole/indolic nitrogen atom (Fig. 4B) and similar ^15^N chemical shift was displayed by indole-based eumelanins^31,33^. In addition, TLC analysis of the degradation products of brown pigment revealed the presence of indole moiety (Fig. S4) confirming the indolic nature of the brown pigment. Presence of high nitrogen and absence of sulfur content further confirms that brown pigment is indole-type melanin and this is in agreement with the previous reports^26^ and also rules out the possibility of brown pigment being DHN or pheomelanin. Interestingly, brown pigment showed similar spectroscopic features of eumelanin (indole-type melanin) derived from tyrosine. In contrast, here for the first time, we identified and characterized indole-type melanin derived from L-tryptophan in a bacterium.

Tyrosine or acetyl CoA derived melanin biosynthesis is well established while Trp-based melanin biosynthesis is a mystery^13–15^. To gain the insights into the Trp-melanin biosynthesis, first, the genome of strain JA2 was mined for the genes of canonical melanin biosynthesis. Interestingly, strain JA2 genome lacks candidate genes for synthesis of DHN, DOPA, and eumelanin except for the pyomelanin pathway genes. Furthermore, biosynthetic inhibitors of DOPA-melanin/eumelanin (Kojic acid)^4^, pyomelanin (Sulcotrione)^18^ and *de novo* tyrosine-based melanin inhibitor glyphosate did not inhibit the Trp-melanin synthesis in strain JA2 (Fig. 5A) suggesting that the Trp-melanin is not synthesized from canonical tyrosine-based melanin biosynthetic process. However, pigment synthesis is inhibited by sodium azide, a laccase inhibitor^4^ and this implies the possible role of laccase in Trp-melanin synthesis. Role of laccase in melanin synthesis is reported in few microorganisms^4,8^, although we did not find gene coding for laccase, we speculate laccase-like enzyme role in Trp-melanin synthesis by strain JA2 as laccase-like enzyme role is reported in melanin synthesis in few bacteria^38,39^.

**Figure 7:**
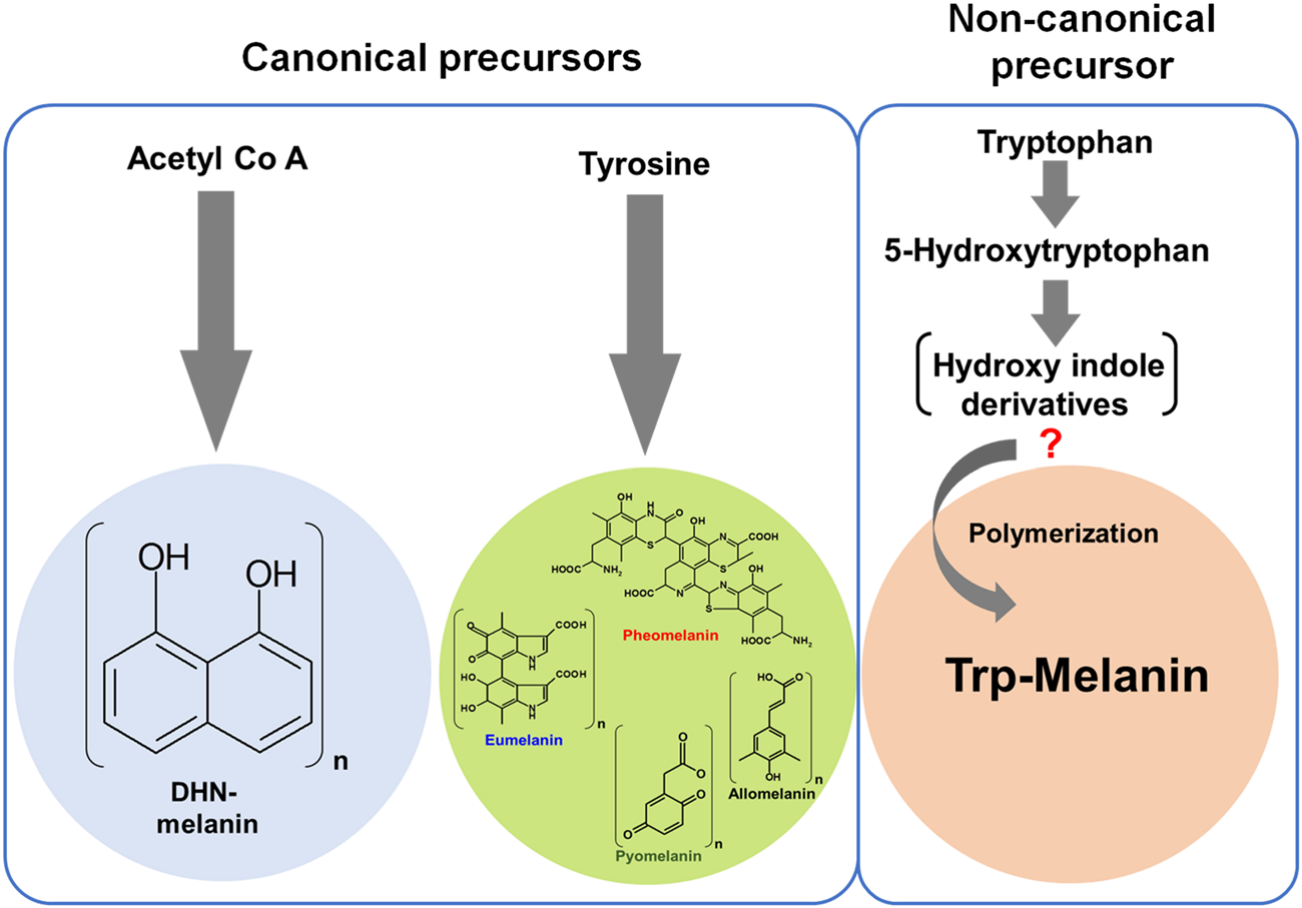
Schematic representation showing melanin synthesis from canonical precursors and non-canonical tryptophan-based melanin synthesis in strain JA2. Predicted trp-based melanin biosynthetic pathway in strain JA2. Metabolites in parenthesis are unknown hydroxyindoles involved in polymerization.

Eumelanin is a polymer of 5,6-dihydroxyindole (DHI) and 5,6-dihydroxyindole-2-carboxylic acid (DHICA), synthesized from tyrosine *via* DOPA, DOPA-chrome finally to DHI, DHICA, and their polymerization lead to eumelanin formation^3,8^. In contrast, for Trp-melanin, tryptophan acts as a precursor and its oxidative metabolism possibly leading to the formation of hydroxyindoles and their subsequent polymerization to melanin (Fig. 7). In support of this, we found OH-Trp and other hydroxyindole derivatives in the exometabolome of Trp-amended aerobic cultures of strain JA2 (Table 1). Hydroxyindole derivatives and brown pigment formation were not found in anaerobic cultures and on the other hand correlation between hydroxyindoles and pigment formation in aerobic cultures indicates possible role of hydroxyindoles in pigment formation. Furthermore, significantly higher brown pigment production in the presence of OH-Trp compared to Trp (Fig. 5B) suggests that OH-Trp may have readily converted to hydroxyindoles and thereby leading to higher pigment production in strain JA2. Taken together these findings suggest oxidative metabolism of Trp leading to the formation of hydroxyindole derivatives and thereby Trp-melanin formation. Similarly, Vekey *et al*. 1992, reported tryptophan-based melanin formation during chemical oxidation of Trp using perchloric acid and study revealed that polymer consists of hydroxyindoles^40^.

However, our study demonstrates oxidative metabolism of Trp leading to Trp-melanin formation in a biological system. Melanins are natural polymers of phenols or hydroxyindoles mostly derived from tyrosine^2,3^ however in our study hydroxyindole derivatives are derived directly from Trp. Interestingly, alkaline chemical oxidation of Trp-melanin revealed presence of PTCA and PTeCA, which are oxidativate products of 5,6-dihydroxyindole-2-carboxylic acid (DHICA)^5,9,34^, thus we speculate that DHICA could be one of the monomer of Trp-melanin. Alternatively, detection of eumelanin markers (PTCA and PTeCA) suggest co-exsitance of eumelanin along with Trp-melanin. Whether DHICA is a involved in Trp-melanin formation needs further investigation, however, the possible role of other hydroxyindole derivatives role in Trp-melanin formation can not be ruled out.

*In vitro* melanin formation by strain JA2 from Trp or its derivative OH-Trp (Fig. 6) and significantly higher pigment synthesis from OH-Trp suggests (Figs. 5B and 6) that OH-Trp is a possible immediate precursor of pigment synthesis. Higher pigment content in the presence of OH-Trp is possible because of availability of high levels of hydroxyindoles derived from OH-Trp and their subsequent polymerization may have resulted in higher melanin synthesis. Previous study on *Planaria* larval eye melanogenesis demonstrated that formation of OH-Trp is essential for pigment synthesis and suggested possible role of OH-Trp in melanogenesis^13^. Similarly, *in vitro* peroxidase, mediated OH-Trp based melanogenesis is reported elsewhere suggesting the role of OH-Trp in melanogenesis^14,15^. Based on findings from the current study we hypothesize Trp-melanin synthesis, wherein during oxidative metabolism, tryptophan, is possibly converted to OH-Trp, thereafter into hydroxyindoles and these subsequently polymerized to melanin (Fig. 7). Although few chemical oxidation and *in vitro* studies revealed Trp-melanin formation, for the first time, our study revealed *in vivo* Trp-melanin synthesis, characterized the melanin and study provided important clues on the possible biochemical route of Trp-melanin synthesis.

In conclusion, our study revealed tryptophan-based melanin synthesis by aerobic cultures of photosynthetic bacterium, *Rubrivivax benzoatilyticus* JA2. Traditionally indole-type melanins (eumelanin) are known to synthesize from tyrosine, however, our study demonstrated novel indole-type melanin (named as Trp-melanin) synthesis from a non-canonical precursor, Trp possibly through an alternative route in strain JA2. Future investigations on the identification of monomeric constituents and transcriptome studies would reveal chemical diversity and possible candidate genes involved in Trp-melanin synthesis in strain JA2. Findings from our study strongly supports the view that tryptophan can be a physiological precursor for melanin synthesis and organisms may have evolved mechanisms to use non-canonical precursors for melanin synthesis based on their physiological conditions. Our study indicates that enigmatic melanin pigment chemical and biosynthetic diversity are far from over and suggests possible occurrence of non-canonical melanin biosynthetic process in other life forms.

## Methods

### Organism, growth conditions and pigment production

*Rubrivivax benzoatilyticus* JA2 (ATCC BAA-35) was used as a model organism for all the experiments and pure culture was maintained and grown photoheterotrophically (under anaerobic condition, pH 6.8, 30 $\pm$ 2 °C; light 2,400 lux) on minimal media containing 22 mM of malate (carbon source) and 7 mM of ammonium chloride as a nitrogen source in completely filled culture bottle (250 ml). Photoheterotrophically grown mid-log phase culture (O.D- 0.4 at 660 nm) was used as inoculum(10%) and culture was grown aerobically in 500/1000 ml conical flasks and incubated at 30±1 °C, 180 rpm in shaker incubator (Innova Eppendorf) for 48 h. Ammonium chloride was replaced with 1 mM of L-tryptophan as a nitrogen source for induction of pigment synthesis. The growth was measured as a change in optical density (OD) at 660 nm, while pigment production was measured spectrophotometrically against uninoculated media as a blank at 400 nm. To check the effect of different melanin inhibitors on brown pigment production by strain JA2, glyphosate (1 mM), sodium azide (1 mM), Kojic acid (0.5 mM) and quercetin (0.15 mM) were added to the aerobic cultures containing L-tryptophan. All the inhibitor studies were carried out with mid-log phase aerobic cultures of strain JA2. Whenever required 1 mM L-tryptophan replaced with 5-hydroxytryptophan(1 mM; Himedia chemicals) as a nitrogen source to check the pigment production.

### Extraction and purification of brown pigment

L-tryptophan amended aerobic cultures of strain JA2 was used to isolate the pigment and after 48 h of incubation, culture was harvested by centrifugation (10,000 rpm for 10 mins at 4 °C). Later the supernatant was collected and acidified to pH 2.0 using 5 M HCl and acidified supernatant was stored at 4 °C for 2–4 days. The brown precipitate settled at the bottom of the flask was collected by centrifugation at 10,000 rpm for 10 mins at 4 °C. The brown pigment was further purified by serial washings using different organic solvents (10 ml of hexane, acetonitrile, acetone, ethyl acetate, ethanol, and methanol) and finally with Milli-Q water. The purified pigment was dried under freeze drier (Labcanco, USA) and dried pigment was used for physicochemical and spectroscopic studies.

### Scanning Electron Microscopic (SEM) and UV visible spectroscopic analysis

SEM analysis of purified pigment was carried out according to Prasuna *et al*.^24^ with slight modifications, In brief, 1 mg of pigment was suspended in 1 ml of acetonitrile, 100 µl of suspended solution or sonicated solution was mounted on a glass slide and fixed on to the stubs. Samples were subjected to gold sputtering and examined using SEM (Philips XL30 series). UV-Visible spectroscopic studies of purified pigment were done on a spectrophotometer (SHIMADZU, Japan) and the spectra were recorded from 200–800 nm using 1 M NaOH as blank.

### FTIR, ESR and X-ray diffraction analysis

FTIR, the spectrum of brown pigment was recorded on alpha ATR-FTIR spectrometer (ALPHA T model, Bruker Optics, Germany) Dried sample was loaded on diamond crystal and the spectrum was recorded from the range of 400–4000 cm^−1^ at resolution 4 cm^−1^ with 20–30 scans in transmittance mode. ESR and XRD analysis of purified pigment was done according to Prasuna *et al*.^18^. Briefly, dried pigment (20 mg) was loaded into the ESR quartz tube and spectra were recorded on JEOL X-band ESR spectrometer at room temperature. X-ray Diffraction spectrum of purified pigment was recorded using Bruker D8 Advance, Germany X-Ray Diffractometer. Spectrum was recorded by scanning the pigment using the CuKα radiation at wavelength 1.5406 A° with a step size of 0.04°, voltage 40 kV, current 40 mA and 2θ range 10–90°.

### Solid-state ^13^C and ^15^N NMR analysis

The Solid-state ^13^C and ^15^N spectra of purified brown pigment were acquired on an Avance III Bruker Spectro spinning as 800 MHz spectrometers operated using as a triple resonance probe with 5 mm. Purified melanin packed (20–30 mg) in ^13^C and ^15^N probe Bruker operating as resonance probe with the operating conditions; spinning speed −7 kHz: 1 H 90°; pulse length was 2.83 μs and the ^13^C 180° pulse length was 6.52 μs. The contact time −2000 μs, acquisition time was 33 ms and the recycle delay-8 s with total 10000 scans. The ^13^C chemical shifts were calibrated relative to tetramethylsilane (0 ppm). The solid-state ^15^N NMR CP/MAS spectrum was obtained with the same instrument using N-15 probe Bruker polarization depends on the ^15^N—H dipole interaction.

### Brown pigment hydrolysis, alkaline oxidation and TLC analysis

The brown pigment degradation products were identified by hydrolysis of the pigment as described by Ellis and Griffiths 1974^41^ with slight modifications. Five milligrams of the dried pure brown pigment was added to 0.5 g of KOH in a 5 ml tightly sealed screw cap glass tube. The mixture was brought to boil on hot water bath (1 h) and the dark color residue thus formed was allowed to cool. To the dried residue 1.5 ml, distilled water was added and acidified (pH 2) with concentrated HCl and metabolites were extracted with diethyl ether and dried under rota evaporator (Heidolph, Germany) finally dissolved in HPLC grade methanol. To detect the indoles, the hydrolyzed fraction was run on TLC (Merk, Silica gel 60 F_254_, 20×10 cm, 0.2 mm,) using a mixture of Chloroform: Methanol: Glacial acetic acid (9: 0.95: 0.05 v/v) as a solvent system and TLC plate was developed using indole-specific TLC reagent prepared as described by Ehmann *et al*.^42^. Alkaline hydrogen peroxide oxidation of brown pigment was carriedout according to Ito et al. 2011 and LCMS analysis was performed as described in HRLC-MS.

### Extraction of metabolites and HRLC-MS analysis

Metabolites were extracted from the tryptophan-amended aerobic cultures of strain JA2 and culture was harvested after 48 h of incubation by centrifugation (10,000 rpm, 4 °C, 10 mins). The supernatant was collected and, the supernatant was dried under vacuum using rotary flash evaporator (Heidolph, Germany). The dried supernatant was dissolved in 5 ml of HPLC grade methanol and centrifuged (12,000 rpm, 5 mins), the clear methanolic fraction was collected and freeze dried under vacuum. Finally, the dried methanolic extract was redissolved in 1 ml MS grade methanol and used for HPLC and LCMS analysis.

HRLC-MS ESI analysis was performed according to Prasuna *et al*.^24^, Briefly, analysis was performed on 6520 Accurate-Mass Q-TOF LC-MS system(Agilent Technologies) equipped with Agilent 1200 series HPLC with a photodiode array detector; an auto sampler was used to inject (2 µl) the sample and chromatographic separation was carried out at 25 °C. Reverse phase column (Phenomenex) C-18 (Luna, 5 µm, 150×4.6 mm) was used to separate the metabolites using a constant flow rate 0.8 ml/minute of mobile phase consisting of 0.1% glacial acetic acid in water (v/v) (eluent A) and acetonitrile (eluent B). The gradient program was used to elute the metabolite (60 mins): starting with 1% eluent B followed by 55% within 48 min then step gradient to 100% within 55 min and held for 5 minutes. The separated analytes were infused into the ESI ion source under full-spectrum scan mode (50–1000 m/z). Data were collected under negative and positive modes in independent runs. Mass Hunter Qualitative software (version 6.0, Agilent Technologies) was used to analyze the raw data. The standards PTCA and PDCA were generously gifted by Prof. Shosuke Ito and Prof. Kazumasa Wakamatsu, Fujita Health University, Japan. 5-hydroxyindole-3-acetic acid standard was purchased from Sigma-Aldrich (Sigma-Aldrich, H8876).

### *In vitro* melanin production by strain JA2

Log phase aerobic cultures grown on L-tryptophan containing media were used for *in vitro* melanin production studies and cell-free extracts were prepared according to Prasuna *et al*.^18^. Briefly, the culture was harvested by centrifugation at 4 °C for 10 min 10,000 rpm, collected pellet was washed thrice with 50 mM Tris-buffer and resuspend in 4 ml of 50 mM (pH 7.5) Tris-HCl buffer. The cell lysate was prepared by sonicating the cells using sonicator (BANDELIN, Germany 8 cycles, 55% amplitude) and cell lysate was centrifuged at 16,000 rpm for 20 minutes. The pellet was discarded, the clear supernatant was collected and was used as an enzyme source for *in vitro* pigment production. The *in vitro* assay was carried out in 4 ml final volume consisting of 1 mM L-tryptophan or OH-Trp as substrate and appropriate amount cell-free extract and reaction mixture was incubated at 180 rpm, 37 °C for 1 hour in a incubator shaker (Innova). The reaction was terminated by adding the 300 µl of concentrated HCl and pre-denatured enzyme (with conc. HCl) and reaction mixture without cell-free extract were used as blanks. After the reaction was terminated the brown pigment was purified as described in material and methods earlier. For time-dependent *in vitro* pigment production studies, same procedure was followed as described above except, 1 ml sample was withdrawn periodically (at 30 mins). Samples were centrifuged and the supernatants were analyzed for Trp or OH-Trp using HPLC.The pigment produced was analyzed spectrophotometrically at 400 nm.

## Supplementary information

Supplemental information.

## Data Availability

Melanin samples and spectral data available from the corresponding author on request.
