# Supplementary material for: Tryptophan, a non-canonical melanin precursor: New L-tryptophan based melanin production by Rubrivivax benzoatilyticus JA2
Source: Sci Rep. 2020 Jun 2;10:8925. doi: 10.1038/s41598-020-65803-6 (PMC7265499; doi:10.1038/s41598-020-65803-6)
Supplement: Supplementary file 1 — Supplemental information. [file 41598_2020_65803_MOESM1_ESM.pdf]

Supplementary information

Tryptophan, a noncanonical melanin precursor: New L-tryptophan based melanin production by *Rubrivivax benzoatilyticus* JA2

Shabbir Ahmad[a], Mujahid Mohammed[a,1], Lakshmi Prasuna Mekala[a,2], Sasikala Chintalapati[b], Ramana Chintalapati[a]*

[a]Department of Plant Sciences, School of Life Sciences, University of Hyderabad, Hyderabad 500046

[b]Centre for Environment, IST, JNT University, Hyderabad 500 085

Present address:

[1]Department of Botany, Bharathidasan Goverment College for Women,

Puducherry U.T. – 605003.

[2]Department of Plant Science, Avvaiyar Government College for Women,
Karaikal, Puducherry- U.T 609 602

***Corresponding author:**

Prof. Ch.V. Ramana, Department of Plant Sciences, School of Life Sciences,

University of Hyderabad, Hyderabad-500 046, Telangana, India.

E-mail: cvramana449@gmail.com;

Tel phone : +91 040 23134502

Fax: +91 040 23010120 & 23010145

Supplementary information Figures

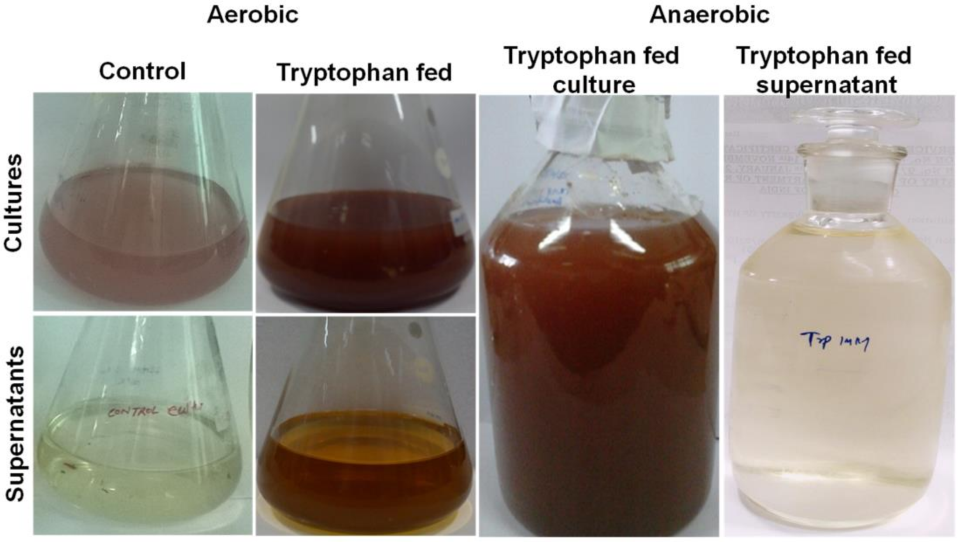

Supporting Information Figure. S1 : Brown pigment production by strain JA2**.** Control (without tryptophan) and tryptophan-amended cultures and respective supernatants of strain JA2 under anaerobic and aerobic growth conditions

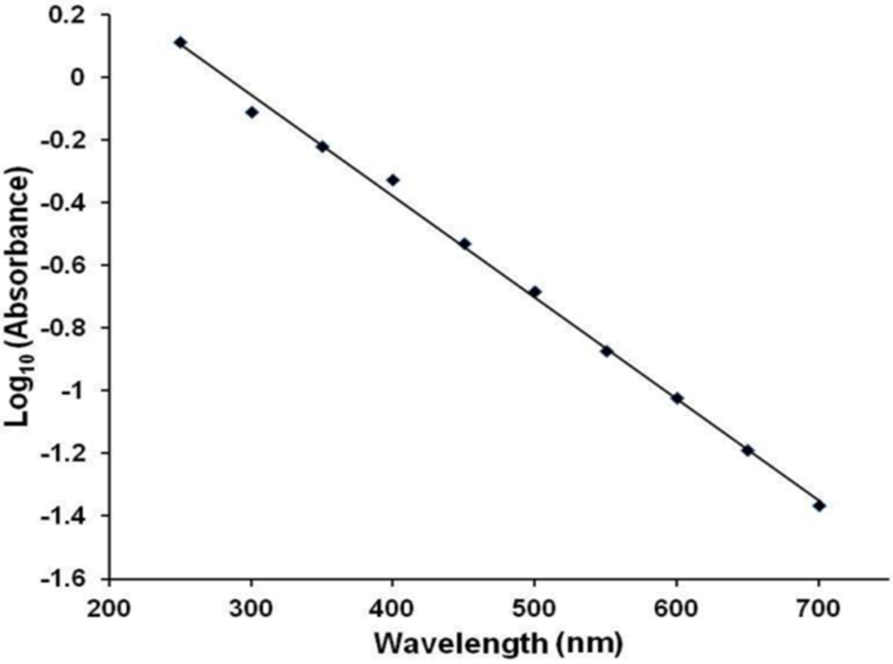

Supporting Information Figure. S2: Linear regression coefficient curve of absorbance vs wavelength of purified brown pigment produced by strain JA2

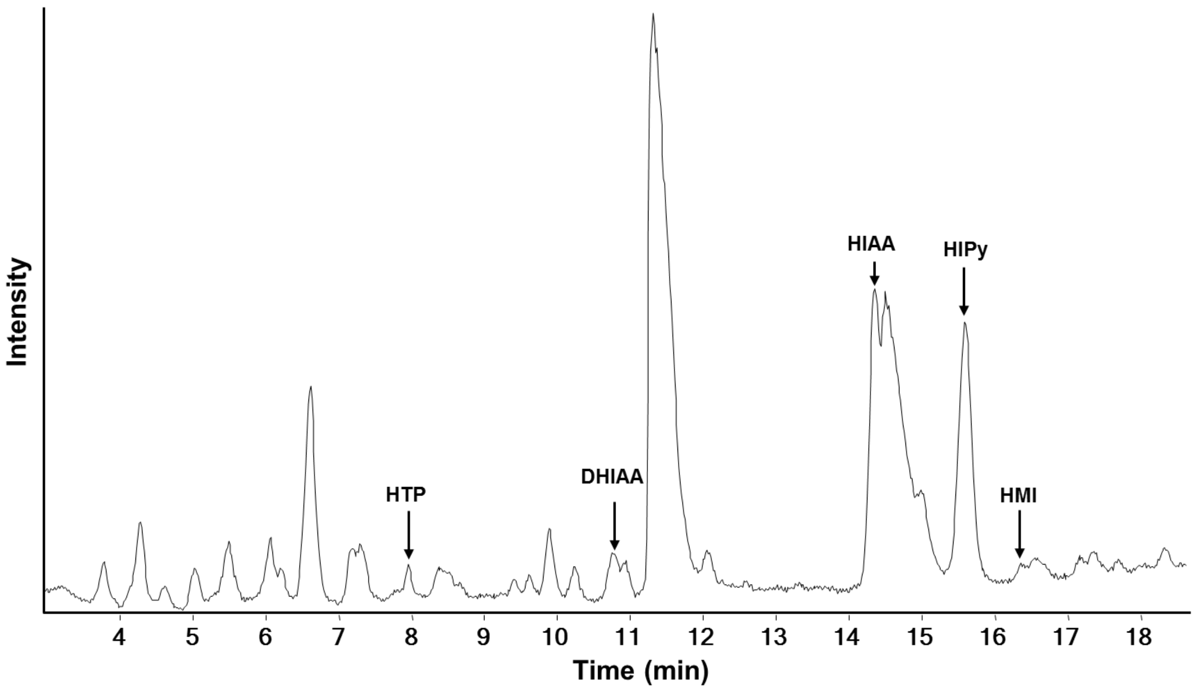

Supporting Information Figure. S3: Total Ion Chromatogram of methanolic extract of tryptophan-amended aerobic culture supernatant of strain JA2. HTP, 5-Hydroxytryptophan; DHIAA, 5,6-dihydroxyindole-3-acetic acid; HIAA, 5-hydroxyindole-3-acetic acid; HIPy, 5-hydroxyindole-3-pyruvic acid; HMI, 5-hydroxymethyl indole.

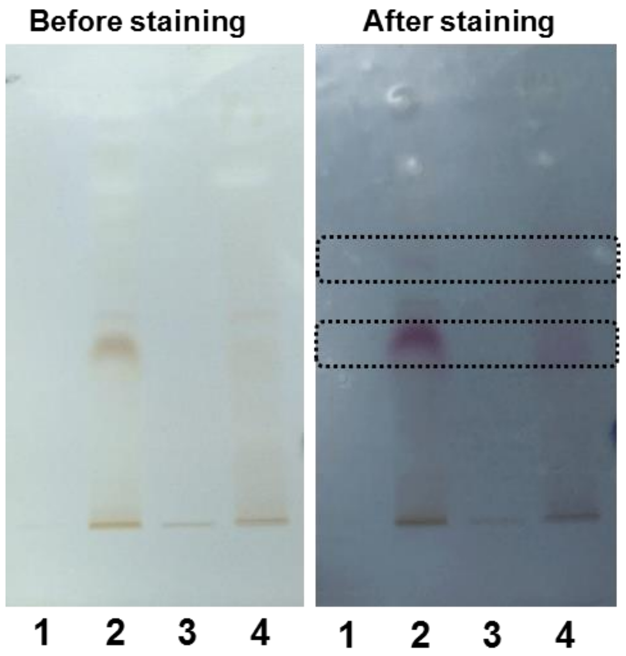

Supporting Information Figure. S4: TLC analysis of hydrolyzed brown pigment products of tryptophan and hydroxytryptophan. Lane-1 and 3: un-hydrolyzed Trp, OH-trp fraction, Lane 2 and 4 hydrolyzed products of Trp and OH-Trp. Rectangular boxes showing the indole positive staining (purple bands

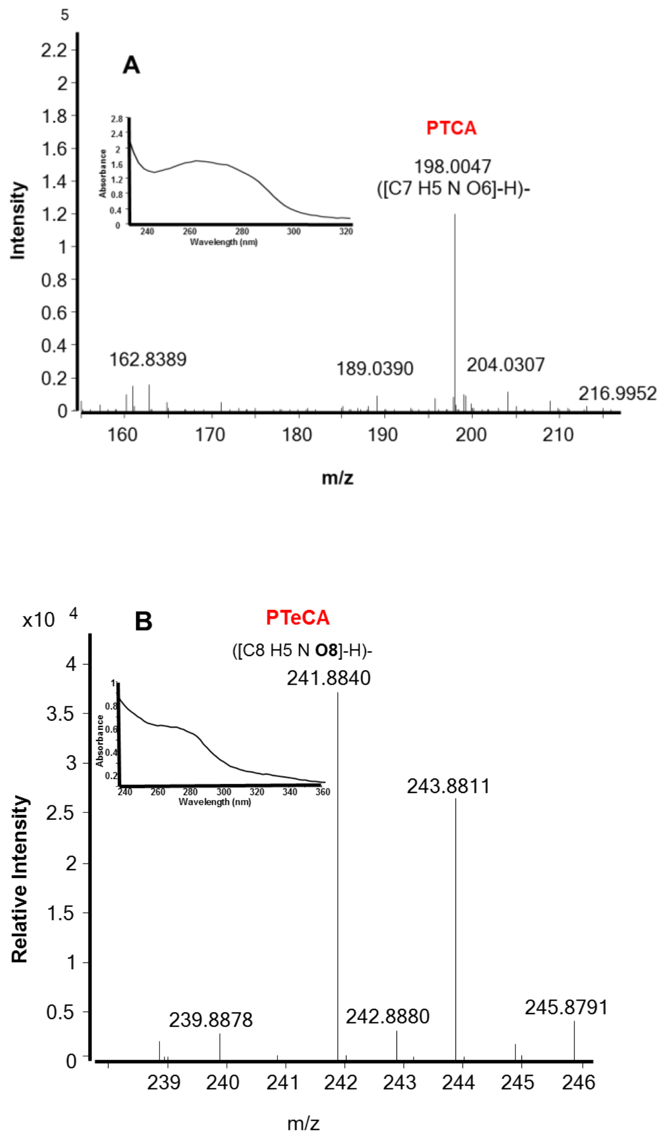

Supporting information Figure S5: Mass spectrum of PTCA (A) and PTeCA showing molecular ion mass of 198 and 241 [M-1] and their corresponding UV spectrum as insert. PTCA, pyrrole-2,3,5-tricaroboxylic acid; PTeCA, pyrrole-2,3,4,5-tetracarboxylic acid.

Supporting Information table. S1: Physicochemical properties of purified brown pigment

	**Chemical nature**	**Result**
1.	Solubility<br>**Organic solvent**<br>(a) Ethanol<br>(b) Chloroform<br>(c) Acetone<br>(d) Benzene<br>(e) Ethyl acetate<br><br>**Alkaline solution**<br>1M NaOH /KOH<br><br>**Buffers**<br>50 mM phosphate buffer pH 7<br>50 mM Sodium bicarbonate buffer pH 10<br>50 mM dipotassium hydrogen phosphate buffer pH 12<br><br>Water	 Insoluble<br>Insoluble<br>Insoluble<br>Insoluble<br>Insoluble<br>Insoluble<br><br><br>Soluble<br><br><br>Insoluble<br>Partially soluble<br>Partially soluble<br><br>Insoluble
(2)	Colour	Dark brown
(3)	Reaction with 3M HCl	Precipitated
(4)	Reaction with oxidizing agent such as H_2O_2 and NaOCl	Decolorized (black to colorless)
(5)	Reaction of polyphenol with $FeCl_3$	Formed flocculent brown precipitate
(6)	Reaction with $Na_2S_2O_4$ and potassium ferricyanide	Decolorized first turned brown with addition potassium ferricyanide
(7)	Reaction with ammonical silver nitrate	Formed a grey coloured silver precipitate (reduced) lining on the test tube
